# Improving Oxygen Conditions in the Deeper Parts of Bornholm Sea by Pumped Injection of Winter Water

**DOI:** 10.1007/s13280-012-0356-4

**Published:** 2012-11-17

**Authors:** Anders Stigebrandt, Ola Kalén

**Affiliations:** Department of Earth Sciences/Oceanography, University of Gothenburg, Box 460, 40530 Gothenburg, Sweden

**Keywords:** Geo-engineering, Oxygenation, Pumping, Oxygen consumption, Vertical diffusivity

## Abstract

Vertical diffusivity and oxygen consumption in the basin water, the water below the sill level at about 59 m depth, have been estimated by applying budget methods to monitoring data from hydrographical stations BY4 and BY5 for periods without water renewal. From the vertical diffusivity, the mean rate of work against the buoyancy forces below 65 m depth is estimated to about 0.10 mW m^−2^. This is slightly higher than published values for East Gotland Sea. The horizontally averaged vertical diffusivity κ can be approximated by the expression κ = *a*_0_ *N* ^−1^ where *N* is the buoyancy frequency and *a*_0_ ≈ 1.25 × 10^−7^ m^2^ s^−2^, which is similar to values for *a*_0_ used for depths below the halocline in Baltic proper circulation models for long-term simulations. The contemporary mean rate of oxygen consumption in the basin water is about 75 g O_2_ m^−2^ year^−1^, which corresponds to an oxidation of 28 g C m^−2^ year^−1^. The oxygen consumption in the Bornholm Basin doubled from the 1970s to the 2000s, which qualitatively explains the observed increasing frequency and vertical extent of anoxia and hypoxia in the basin water in records from the end of the 1950s to present time. A horizontally averaged vertical advection–diffusion model of the basin water is used to calculate the effects on stratification and oxygen concentration by a forced pump-driven vertical convection. It is shown that the residence time of the basin water may be reduced by pumping down and mixing the so-called winter water into the deepwater. With the present rate of oxygen consumption, a pumped flux of about 25 km^3^ year^−1^ would be sufficient to keep the oxygen concentration in the deepwater above 2 mL O_2_ L^−1^.

## Introduction

The deepwater of the Baltic Sea is replenished with sea salt and oxygen by new deepwater with enhanced salinity coming from Kattegat and flowing through the Arkona and Bornholm Seas. The lower layers of the Bornholm Sea are flushed by new deepwater of salinity in the range 13–20 that is entering essentially through the Bornholm Strait. Inflow of new deepwater to the Arkona Sea occurs when the sea level stands higher in Kattegat than in the Arkona Sea. Inflows are often described as episodic events and the largest events occur when the sea level in the Baltic starts from a low level and the sea level in Kattegat stays high for a couple of weeks. During the so-called major inflows amounting to 150–300 km^3^, occurring only once or twice during a decade, the sea level in the Baltic Sea rises 0.5–1 m (Schinke and Matthäus 1998; Gustafsson and Andersson 2001).

In the beginning of an inflow event, the salinity of the inflowing water is rather low since a large fraction of the initial water is old surface water from the Baltic proper that has spent only short time in the Belt Sea and Kattegat before flowing back again. During this short time little mixing has occurred with the underlying saltier water. As the inflow proceeds, however, the salinity of the inflowing water increases. Thus, the mean and maximum salinities of new deepwater are greater for larger than for smaller inflows. When flowing through the Arkona Sea along the bottom as a dense current, the new deepwater mixes with residing water. When entering the Bornholm Basin, the volume flow has increased by about 60 % and the salinity has decreased accordingly (Stigebrandt 1987a). Additional mixing occurs before the new deepwater reaches the deepest part of the Bornholm Basin where the highest observed salinity exceeds 18.

Only the densest new deepwater may reach down to the greatest depth in the Bornholm Sea. Less dense inflows are interleaved in the halocline (pycnocline) usually in the depth range 50–70 m. High-saline water from large inflows may reside for several years in the deeper parts of the Bornholm Sea, which has a maximum depth of about 100 m. The Stolpe Channel, with sill depth 59 m, permits halocline water to exit the Bornholm Basin and flow into the East Gotland Sea.

The frequency of exchange of basin water is generally determined by the density range of the new deepwater and the rate of vertical density reduction in the basin due to vertical mixing. A large density range of the new deepwater tends to give long residence times in the deeper parts of the basin water, while large rates of vertical mixing tend to give short residence times as explained in Stigebrandt (2012). The frequency of occurrence as well as the vertical extent of hypoxia and anoxia in the basin water increase with both the residence time of the basin water and the rate of supply of organic matter.

In this paper we show that the rate of density reduction in the basin water may be increased artificially by forced mixing, e.g., by pumping less dense water from higher strata into the basin water. This will lead to an increased frequency of water exchange and a decreased residence time of basin water. In addition, pumping will also lead to an increased supply of oxygen to the deepwater. Both effects will raise the minimum concentration of oxygen in the basin water as demonstrated by our model simulations. Raising the oxygen content and decreasing the density of the Bornholm Sea basin water would imply that the Baltic proper east of Bornholm Sea will receive water of higher oxygen concentration and lower salinity during the so-called major inflows which would help to improve the oxygen conditions in the Baltic proper. This paper is structured as follows: we first describe the oceanographic conditions in Bornholm Sea. Then a description of the theory used for computations in this paper is given and finally we show the results for our computations of vertical diffusivity and oxygen consumption in the basin water with the pumping applied, followed by a discussion.

### Topography and Hydrography of the Bornholm Sea

**Fig. 1:**
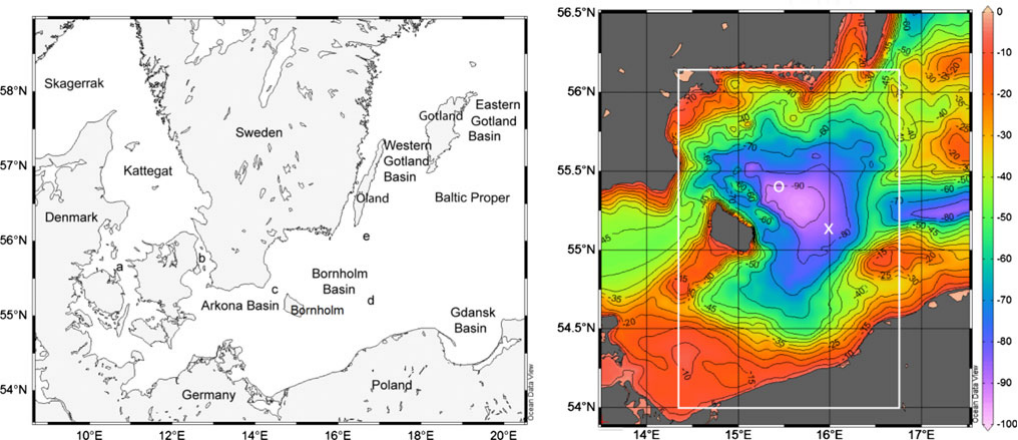
*Left panel* Map of the Baltic Sea. Names mentioned in the paper are shown in the map; *a* The Great Belt, *b* Öresund, *c* Bornholm Channel, *d* Stolpe Channel, *e* Öland Channel. *Right panel* A close-up showing the bathymetry, using the spherical grid topography of the Baltic Sea by Seifert et al. (2001). The locations of the monitoring stations BY4 and BY5 are indicated by the *white circle* and *cross*, respectively. The domain of the advection–diffusion model is given by the *white rectangle*

The Bornholm Sea, situated east of the island of Bornholm, has a maximum depth of about 100 m. In the east it is connected to East Gotland Sea by Stolpe Channel, with a maximum depth of about 59 m, see the topographical map in Fig. 1. There is also a 46 m deep connection to the West Gotland Basin by a trench southeast of Öland. The Bornholm Channel, northwest of Bornholm, is the main inlet of new deepwater to Bornholm Sea. It is about 40 m deep in the west with increasing depth towards the east. A submarine ridge, with maximum depth of about 29 m, running towards southwest from Bornholm provides the western border between the Bornholm and Arkona Seas (Fig. 1). Most of the new deepwater from the Arkona Sea enter the Bornholm Sea through the Bornholm Channel. Only during the so-called major inflows some new deepwater may spill across the shallower southern ridge (Stigebrandt 1987a; Lass et al. 2001).

The spherical grid topography of the Baltic Sea by Seifert et al. (2001) was used to compute the hypsographic function, i.e., the horizontal area of the basin at different depths. The basin used for the computations is the rectangular region bound by the coordinates 54.00°N, 14.25°E and 56.21°N, 16.75°E, shown by the white rectangle in Fig. 1. The horizontal surface area of Bornholm Basin is about 14 150 km^2^ at sill level (59 m) and the volume below the sill is about 200 km^3^.

Hydrographical observations of temperature, salinity, and oxygen concentration from the period 1957 to 2011 from the monitoring stations BY4, located at 55.38°N, 15.33°E, and BY5 at 55.25°N, 15.98°E, see Fig. 1, were used for this study. The numbers of unique dates of measurements used in the series from BY4 and BY5 were 352 and 433, respectively.

**Fig. 2:**
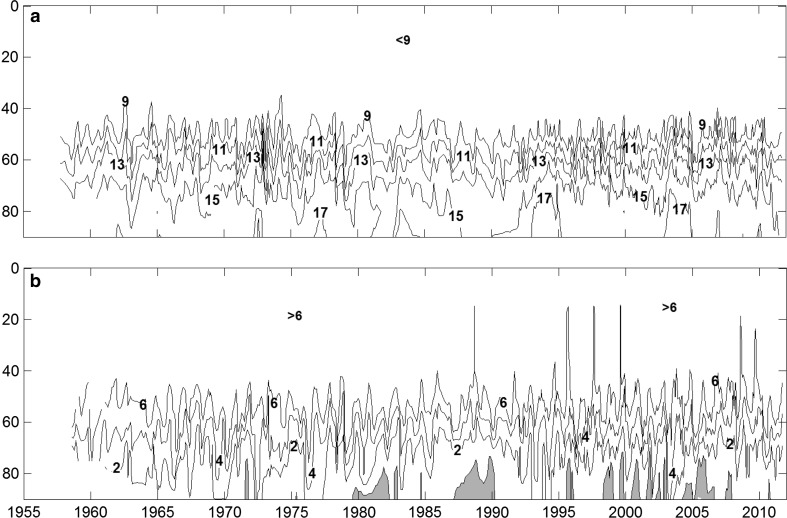
Isopleth diagram of salinity (**a**) and oxygen (mL L^−1^) (**b**) at BY5 from 1957 to 2010

The salinity in the Bornholm Sea is rather constant from the surface down to the halocline from where it increases rapidly with depth (Fig. 2a). The vertical location of the halocline is influenced by the depth of the Stolpe Channel. This is the reason why the halocline is closer to the sea surface in Bornholm Sea than in the main part of the Baltic proper. The top of the halocline stands highest in connection with major inflows of new deepwater.

The isopleth diagram for salinity at BY5 (Fig. 2a) shows that the salinity in the deeper part of the basin increases rapidly in a number of events due to inflow of new dense deepwater. Between these events the salinity decreases slowly due to vertical diffusion. This suggests that the residence time of water in the lower part of the basin can be several years. This impression is strengthened by the isopleth diagram of oxygen (Fig. 2b), showing that deepwater renewals increase the oxygen concentration in the deepwater, but after a few years the oxygen is exhausted and anoxia occurs.

Since the beginning of the 1980s, the oxygen concentration is often less than 2 mL L^−1^ (1 mL ≈ 44.6 μmol) below 70 m depth. Anoxia seems to have occurred at BY5 for the first time about 1980 and since then on several occasions. Oxygen diffusion from above is obviously not sufficient to supply all the oxygen needed for respiration at the greatest depths. This suggests that oxygen consumption by respiration has increased rapidly from the 1960s to the 1980s.

## Materials and Methods

### Vertical Mixing and Buoyancy Fluxes in the Basin Water

**Fig. 3:**
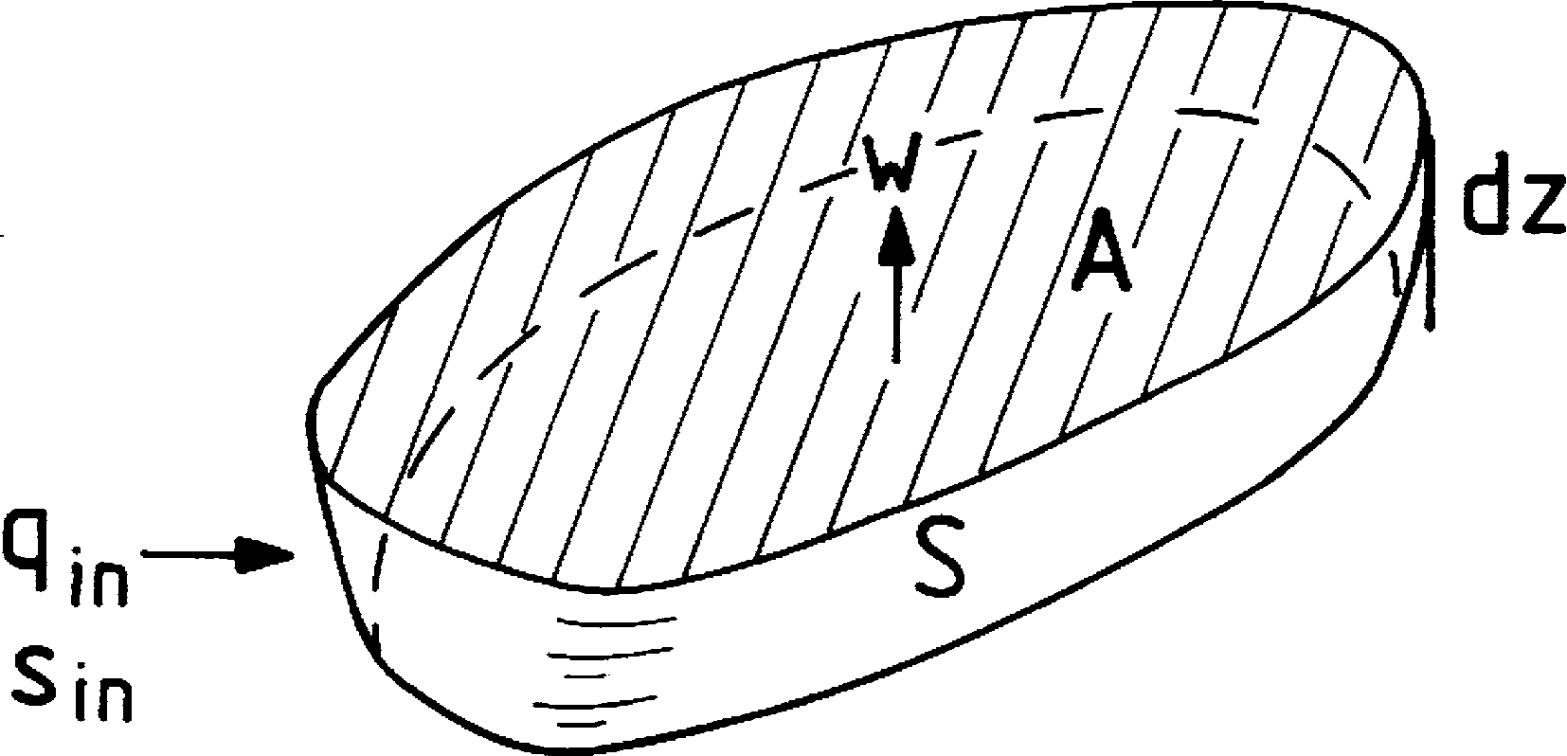
A horizontal slice (slab) of a fjord basin centered at depth *z*

The water of isolated basins may be treated as one-dimensional if horizontal gradients are much smaller than vertical. We first consider circulation caused by vertical mixing and inflows of new deepwater. The one-dimensional vertical advection–diffusion equation for salt is then an appropriate starting point. Consider a horizontal slice (slab) of a basin centered at the depth *z* (Fig. 3). The slice has thickness d*z*, horizontal surface area *A*(*z*), volume *V*(*z*) = *A*(*z*)d*z*, and salinity *S* = *S*(*z*). Water exchange with neighboring basins introduces flows *q* = *q*(*z*) into and out of the basin which in turn introduce vertical advection of velocity *w*(*z*). The salinity of water flowing into the slab by the flow *q*(*z*) is *S*_in_(*z*). For horizontal flow out of the slab (*q* is negative) *S*_in_ equals *S*(*z*). Salt is also transported through the horizontal boundaries of a slab due to vertical turbulent diffusion.

If the vertical *z*-coordinate is positive upwards, conservation of salt in the slab is described by

1 $$ \frac{\partial S}{\partial t} = \frac{1}{A}\left( {\frac{\partial }{\partial z}\left( {A\kappa \frac{\partial S}{\partial z}} \right) - \frac{\partial }{\partial z}\left( {wAS} \right) + qS_{\text{in}} } \right). $$

Conservation of volume requires that

2 $$ q - \frac{\partial }{\partial z}(wA) = 0. $$

If non-conservative substances like dissolved oxygen are considered, there must be a sink term in the equation as discussed below. The vertical advection–diffusion model above is discussed in, e.g., Stigebrandt (1987b, 1990).

Observations in fix points show turbulence as high-frequency fluctuations of, e.g., velocity, salinity, temperature, and other state variables. The vertical flux of, for instance, sea salt may be estimated as the time average of the product of the fluctuating components *w*′ and *S*′ of the vertical velocity and the salinity, respectively. It is common to describe the turbulent vertical flux of a scalar as a product of the vertical turbulent or eddy diffusivity κ and the vertical gradient of the scalar. For a given locality, it is reasonable to assume that the same value of κ may be used to compute vertical turbulent transports of different scalars. Thus, for vertical fluxes of salt *S* and temperature *T* one obtains

3 $$ \kappa \cdot \frac{\partial S}{\partial z} = \overline{{w^{\prime } S^{\prime } }} $$

4 $$ \kappa \cdot \frac{\partial T}{\partial z} = \overline{{w^{\prime } T^{\prime } }} $$

Similar expressions can be formulated for vertical fluxes of other scalars.

Vertical fluxes of buoyancy tend to change the stratification and have crucial dynamical significance. Buoyancy is denoted *g*′ = *g*ρ′/ρ_0_, where *g* is the acceleration of gravity, ρ′ the fluctuating part of the density and ρ_0_ a reference density. An equation for the turbulent flux of density $\overline{{w^{\prime } \rho^{\prime } }}$ may be obtained using the equation of state for seawater

5 $$ \rho = \rho_{0} ( 1- \alpha T + \beta S) $$

Here ρ is density, α the coefficient of heat expansion, and β the coefficient of salt contraction. By multiplying Eq. (3) and Eq. (4) by ρ_0_β and ρ_0_α, respectively, and using Eq. (5), the following equation may be obtained after some algebraic operations

6 $$ \kappa \cdot \frac{\partial \rho }{\partial z} = \overline{{w^{\prime } \rho^{\prime } }} $$

The buoyancy flux $b = \overline{{w^{\prime } g^{\prime } }}$ is obtained if Eq. (6) is multiplied by *g*/ρ_0_, thus

7 $$ \overline{{w^{\prime } g^{\prime } }} = \kappa \cdot N^{2} $$

Here *N* ^2^ = −(*g*/ρ_0_)·(∂ρ/∂*z*) is the buoyancy frequency. The buoyancy flux *b* also expresses the rate of work against the buoyancy forces (per unit mass, i.e., W kg^−1^) performed by mixing processes in stratified fluids.

During stagnant conditions in closed basins, changes of salinity and other conservative substances occur exclusively due to vertical diffusion, i.e., *q* = 0 and *w* = 0. The horizontally averaged diffusion equation for salt, Eq. (1), then reads

8 $$ \frac{\partial S}{\partial t} = \frac{1}{A}\left( {\frac{\partial }{\partial z}A\kappa \frac{\partial S}{\partial z}} \right) $$

Here *A*(*z*) is the horizontal area of the basin.

In this case horizontally averaged values of κ may be estimated from horizontally averaged vertical profiles measured at two different occasions. Vertical integration of Eq. (8) from the greatest depth *z* = *d*, where there is no diffusive flux of mass (through the sea bottom), to some level *z* = *u*, gives the following expression for the vertical diffusivity at the level *z* = *u*:

9 $$ \kappa_{z = u} = \left( {A\frac{\partial S}{\partial z}} \right)_{z = u}^{ - 1} \int\limits_{d}^{u} {\frac{\partial S}{\partial t}A{\text{d}}z} $$

This equation may be used to compute κ at *n* − 1 levels if there are *n* levels of measurements of *S* = *S*(*z*,*t*). To obtain significant changes of *S*(*z*,*t*), the time interval between the measurements usually must be of the order of several weeks in order to get significant changes of the state variables. This method to estimate horizontally averaged κ, the so-called budget method, is described at length by, e.g., Gargett (1984). Note that estimates of κ using the budget method are true horizontal averages. They are independent of the value of the coefficient of mixing efficiency *Rf* (the flux Richardson number), which is not the case for estimates of κ using observations of dissipation.

Application of the budget method to cases where all the small-scale turbulence is located in a thin layer near the bottom (boundary mixing) is discussed in Stigebrandt (1976). The budget method to estimate the vertical diffusivity has been applied to numerous fjord basins, e.g., Stigebrandt and Aure (1989) and to the Baltic proper by, e.g., Axell (1998) and Gustafsson and Stigebrandt (2007).

The total rate of work against the buoyancy forces, *P*_B_, by mixing processes below the level *z* = *h* (*h* deeper than the sill level) in a basin is obtained by integrating the buoyancy flux, *b* = κ*N* ^2^, from the greatest depth *z* = *d* to the level *z* = *h*:

10 $$ P_{\text{B}} = \int\limits_{d}^{h} {\rho_{0} \kappa (z)N^{2} (z)A(z){\text{d}}z} $$

Thus, *P*_B_ can be computed from Eq. (10) if the horizontal mean diffusivity κ(*z*) has been determined. To compare the buoyancy fluxes in various basins, one may divide *P*_B_ by the area of the basin at the upper integration limit *h*. This gives the normalized power *W*(*h*) = *P*_B_/*A*(*h*) spent to buoyancy fluxes in the basin water beneath the depth *h*.

### Oxygen Consumption in the Basin Water

One may apply the budget method also to biologically active substances like nutrients and oxygen if a sink (or source) term is added to Eq. (8), e.g. Gustafsson and Stigebrandt (2007). If *c*_m_ is the spatial mean concentration of the substance in the volume *V* below *z* = *u* and *Φ* is the specific source (or sink), expressed as a flux per unit horizontal surface area, one obtains

11 $$ V\frac{{{\text{d}}c_{\text{m}} }}{{{\text{d}}t}} = \left( {\kappa A\frac{{{\text{d}}c}}{{{\text{d}}z}}} \right)_{z = u} + A(z = u)\Upphi $$

In Eq. (11), changes of the inventory are due to both sources (sinks) and vertical diffusion through the upper horizontal surface at *z* = *u*. To apply the equation, the vertical diffusivity κ at *z* = *u* must be estimated by using Eq. (9) on the observed concentrations of sea salt or another conservative substance.

### Density Reduction by Pumping Less Dense Winter Water into the Basin Water

If water flowing into the basin is denser than resident basin water, it will form a dense plume-like bottom current. This entrains ambient water at the rate −*q*, i.e., there is an outflow from the slab to the plume, whereby the volume flow of the plume increases with depth.

The combination of Eqs. (1) and (2) gives the following equation for the rate of change of salinity of a slab in a basin with a dense bottom current:

12 $$ \frac{\partial S}{\partial t} = \frac{1}{A}\left[ {\frac{\partial }{\partial z}\left( {A\kappa \frac{\partial S}{\partial z}} \right) + wA\frac{\partial S}{\partial z} + q(S_{\text{in}} - S)} \right] $$

This can also be applied to a basin with a vertical buoyant plume induced by pumping. Then *q* is the flow out of/into the slab caused by the entraining plume. At depths where the plume entrains ambient water, *q* is negative whereas at the depth where the plume is interleaved into the fjord basin, *q* is positive.

The plume flow is computed as follows. A certain volume flow *Q*_0_ is pumped from the level *z* = *u* down to *z* = *l* where it is discharged in the form of horizontal jets. A certain entrainment flow *q*(*l*) is initiated by the kinetic energy of the jets:

13 $$ q(l) = \alpha \cdot Q_{0} $$

The entrainment coefficient α depends on the kinetic energy of the jets and is typically in the interval (0 < α < 10).

The time step Δ*t* used in the computation is not allowed to be so large that more than half of the water in a layer is entrained, thus

14 $$ \Updelta t \cdot q\left( z \right) < A\left( z \right) \cdot \Updelta z/ 2 $$

Here Δ*z* is the vertical resolution of the computational grid so that *A*(*z*)·Δ*z* is the volume of the slab.

The flow of the plume after the initial mixing equals

15 $$ Q = Q_{0} (1 + \alpha ) $$

The salinity SP and temperature TP of the plume after initial mixing equals

16 $$ {\text{SP}}\left( l \right) = \left( {S\left( u \right) + \alpha S\left( l \right)} \right)/(1 + \alpha ) $$

17 $$ {\text{TP}}\left( l \right) = \left( {T\left( u \right) + \alpha T\left( l \right)} \right)/(1 + \alpha ) $$

If the density of the plume ρ*P*(*l*) is less than the density of the water in the level next above, i.e., ρ*P*(*l*) < ρ(*l* − 1), the plume will move upwards under entrainment and the entrained flow equals

18 $$ q(z - 1) = E \cdot Q $$

Here *E* is the coefficient of entrainment for buoyancy plumes. The new salinity and temperature of the plume when reaching the level *z* − 1 are given by

19 $$ {\text{SP}}\left( {z - 1} \right) = \left( {{\text{SP}}\left( z \right) + {\text{ES}}\left( {z - 1} \right)} \right)/(1 + E) $$

20 $$ {\text{TP}}\left( {z - 1} \right) = \left( {{\text{TP}}\left( z \right) + {\text{ET}}\left( {z - 1} \right)} \right)/(1 + E) $$

If the density of the plume is less than the density of the ambient water on the next level above (*z* − 2) convection continues. Otherwise the plume is interleaved at the level *z* − 2. Note that the water volume to interleave *Q*_int_ equals

21 $$ Q_{\text{int}} = \mathop \sum \limits_{l}^{t} q(z) + Q_{0} $$

The value of *q*(*z*) is now known and new salinities and temperatures of the ambient basin water may be computed using Eq. (12).

If the model is used to simulate the evolution of a non-conservative substance the appropriate source (sink) has to be added to Eq. (12), as discussed earlier. In the present paper, a simplified description of the jet and plume using constant values of α and *E* is used for simplicity.

## Results

The budget methods to estimate the vertical diffusivity and the rate of oxygen consumption described above, require stagnation periods when vertical advection is negligible. To make sure that no inflow of dense water by a bottom current has occurred during the time between two consecutive measurements, two criteria were used. The first is that the salinity at the greatest depth must decrease with time, ∂*S*/∂*t* < 0, owing to vertical diffusion. An inflow of dense water would increase the density and likely also the salinity of the basin water. During times of no inflow, the consumption of oxygen by biological and geochemical processes will decrease the content of oxygen in the basin water. Accordingly, the second criteria for a stagnation period with negligible vertical advection is that the oxygen concentration should decrease with time, ∂O_2_/∂*t* < 0. In our analysis of data from Bornholm Sea we require that both conditions are satisfied.

### Vertical Diffusivity and Buoyancy Fluxes

The vertical diffusivity κ was estimated for stagnation periods as described above. It was determined for the volume below depths *h* equal to 65, 75, and 85 m, respectively. Calculations were done using data from BY4 and BY5 separately. As expected, the results using data from these two stations are quite similar because observations are from the same water mass, see Fig. 1. Average vertical diffusivity at the three levels is given in Table 1. From the vertical diffusivity and the actual vertical stratification we have computed the total rate of work against the buoyancy forces by mixing processes, *P*_B_, using Eq. (10). In Table 1 we give *W* = *P*_B_/*A*(*h*) which is the normalized work against the buoyancy forces below the depth *h*.

**Table 1:** Averages and standard deviations of vertical diffusivity κ (m^2^ s^−1^) at depth *h* (m), work *W* (W m^−2^) against the buoyancy forces below the depth *h*, *a*_0_ (m^2^ s^−2^) (see Eq. 10) at the depth *h* and oxygen consumption O_2_ Cons (g m^−2^ year^−1^) below the depth *h* in the Bornholm Sea based on hydrographical data from BY4 and BY5. No. κ is the number of estimates of vertical diffusivity and No. O_2_ is the number of estimates of oxygen consumption

*h*	κ	No. κ	*W*	*a*_0_	O_2_ Cons	No. O_2_
65	2.5 ± 1.7 × 10^−6^	12	1.0 ± 0.5 × 10^−4^	1.1 ± 0.8 × 10^−7^	74 ± 42	11
75	4.5 ± 3.6 × 10^−6^	35	0.7 ± 0.5 × 10^−4^	1.3 ± 0.9 × 10^−7^	77 ± 61	31
85	8.0 ± 7.2 × 10^−6^	88	0.4 ± 0.4 × 10^−4^	1.4 ± 1.3 × 10^−7^	50 ± 40	85

The variability of all quantities in time is quite large which mirrors how the turbulent activity is forced by the wind which varies between different stagnation periods. There is a clear minimum in *W*(85) in summer, 37 versus about 57 μW m^−2^ during the other seasons (not shown), supporting the conclusion by Axell (1998) that the wind is the major energy source for the turbulence beneath the halocline. It is not clear by which mechanisms energy is transferred to turbulence in the basin water, c.f. the discussion at the end of this paper.

It is common to relate κ to *N* by the following relationship:

22 $$ \kappa = a_{0} N^{ - 1} $$

Here *a*_0_ is an empirical intensity factor (velocity squared) accounting for the mean mixing activity of turbulence. Using a vertical advection–diffusion filling-box model with an entraining dense bottom current carrying new deepwater into the basin, Stigebrandt (1987b) estimated *a*_0_ = 2.0 (± 0.7) × 10^−7^ (m^2^ s^−2^) for the Baltic proper. Modern circulation models for the Baltic proper use κ described by Eq. (22) with *a*_0_ = 1.5 × 10^−7^ (m^2^ s^−2^) (e.g., Meier 2001; Gustafsson 2003; Omstedt 2011).

### Oxygen Consumption

Oxygen consumption was estimated for stagnation periods, the number of such periods increases with depth and is 11 for the volume below 65 m and 85 for the volume below 85 m, see Table 1. The average rate of oxygen consumption is about 75 g O_2_ m^−2^ year^−1^, which corresponds to oxidation of about 28 g C m^−2^ year^−1^. However, there are large variations between the estimates as shown by the large standard deviation. Our analysis for the volume below 85 m shows that the oxygen consumption in the period 1970–1979 is 27.2 g m^−2^ year^−1^ increasing to 58.0 g m^−2^ year^−1^ in the period 2000–2009. The increasing frequency of anoxia from the end of the 1950s to the present, Fig. 2, qualitatively confirms that there is a trend with increasing oxygen consumption.

### Model Simulation of a Stagnation Period

**Fig. 4:**
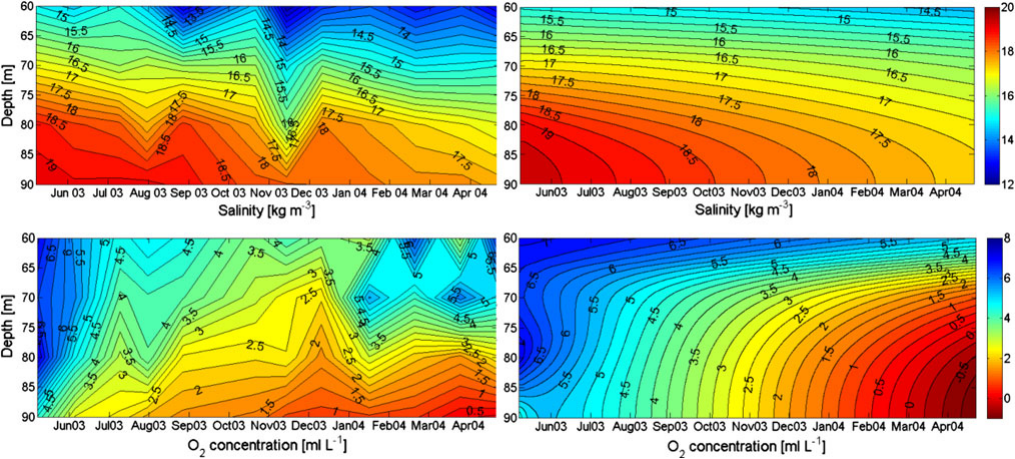
*Upper panels* show observed (*left*) and modeled (*right*) salinity in the basin water of the Bornholm Sea from 7 May 2003 to 21 April 2004. *Lower panels* show observed (*left*) and modeled (*right*) evolution of oxygen (mL L^−1^) during the same period

**Fig. 5:**
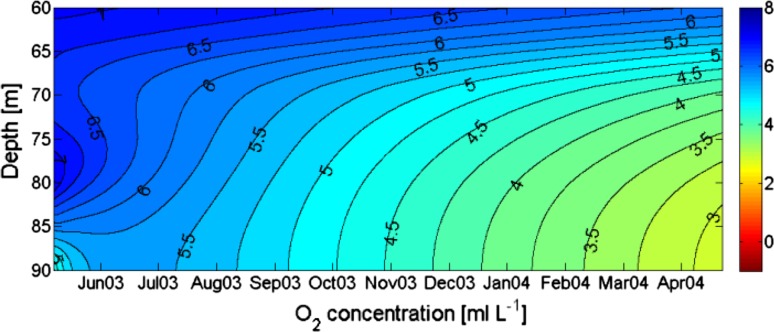
Evolution of oxygen concentration (mL L^−1^) when oxygen consumption was halved as compared to normal consumption. This should be representative for the 1960s and 1970s

The vertical advection–diffusion model described above with the state variables salinity, temperature, and oxygen was applied to the lower parts of the Bornholm Sea. Vertical diffusivity was computed using Eq. (22) with *a*_0_ changing with depth according to Table 1. The vertical variation of the oxygen consumption applied in the model is according to observations given in Table 1. The model was run for a stagnation period in the basin water that started with unusually high salinity. Figure 4 (upper panels) shows the development of the salinity as observed (left) and according to the model run (right). The observations show a large oscillation in November 2003 but there does not seem to be any exchange of water in the lower layers. The salinity in the model develops approximately as in the observations. Figure 4 (lower panels), shows that the evolutions of observed and modeled oxygen are rather similar. A simulation with halved oxygen consumption, intended to show conditions before the 1980s, shows high oxygen concentrations at the end of the simulation period (Fig. 5). This is qualitatively in accordance with observations from that period.

### Changing Hydrography and Oxygen Conditions by Pumping

**Fig. 6:**
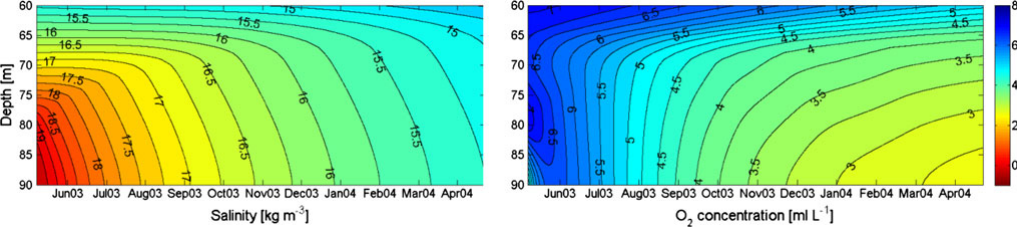
Computed evolution of salinity (*left panel*) and oxygen (mL L^−1^) (*right panel*) in the basin water of Bornholm Sea when pumping 800 m^3^ s^−1^ from 40 to 90 m depth. *E* = 0.05 and α = 10

We then implemented a pump in the model that takes water from 40 m depth, where there is always cold so-called winter water present, and releases it as horizontal jets at 90 m depth. The initial entrainment by the jets is ten times larger than the pumped flow (α = 10). The value of the entrainment factor *E* = 0.05 is adopted from a similar pumped flow in Byfjorden (Liljebladh and Stigebrandt, unpubl.). For a pumped flow *Q* = 800 m^3^ s^−1^, Fig. 6 shows that the minimum oxygen concentration equals about 2.75 mL O_2_ L^−1^ and the salinity is about 15.25 after slightly less than 1 year of pumping. The rate of salinity reduction approximately doubles due to the pumping and at the same time the diffusion of oxygen increases so that the rate of change of oxygen is similar to that in the 1960s (c.f. Fig. 5) a time period when the observed oxygen conditions always were good. In the model, we have not implemented any inflow of new deepwater due to the reduction of the density of the basin water. However, an inflow would likely appear in the later part of the pumping period because the deepwater salinity almost never reaches such low values as 15.25 before an inflow occurs, c.f. Fig. 2. It is therefore suggested that this rate of pumping should keep the basin water well oxygenized with the contemporary rate of oxygen consumption.

## Discussion

The time-averaged work against the buoyancy forces in the basin water of the Bornholm Sea, 100 μW m^−2^ is about twice the value for the East Gotland Sea and slightly less than half the value for Landsort Deep as determined by Axell (1998). In all three locations the work against the buoyancy forces has a seasonal cycle with minimum values in summer which should be a typical feature of mixing processes driven by the wind. Since the mean thickness of the basin water below 65 m equals 12 m, the mean rate of dissipation in the basin water equals (1.2 × 10^−8^)·(1 − *Rf*)/*Rf* W kg^−1^ where *Rf* is the fraction of the turbulent energy that is used for vertical mixing and (1 − *Rf*) is the fraction that dissipates to heat. With *Rf* = 0.1 the mean dissipation equals 1.08 × 10^−7^ W kg^−1^ which is about one order of magnitude greater than the dissipation by interior mixing obtained by van der Lee and Umlauf (2011) in Bornholm Sea. This suggests that most of the mixing occurs at the boundaries (boundary mixing). Dominance of boundary mixing over interior mixing in the East Gotland Sea was demonstrated by Holtermann et al. (2011) and Holtermann and Umlauf (2012). The latter authors discussed mechanisms that transfer energy to the turbulence in the basin water and pointed to the importance of sub-inertial motions (topographic waves). Liljebladh and Stigebrandt (2000) estimated that inertial currents in the surface layer may generate super-inertial internal gravity waves that transfer some 0.3 mW m^−2^ to the deepwater. If the efficiency of turbulence with respect to creating buoyancy fluxes equals 0.1 (i.e., *Rf* = 0.1), the internal waves should contribute a work against the buoyancy forces amounting to 30 μW m^−2^, which is about 30 % of the work as estimated above. Nohr and Gustafsson (2009) computed the energy transfer from wind-forced barotropic currents to baroclinic waves at topography by the same mechanism that generates internal tides which are known to transfer most of the energy needed by deepwater turbulence in the ocean and in fjords (e.g., Stigebrandt and Aure 1989). This mechanism may explain the large variations in work against the buoyancy forces between basins, which depend on the topography and the amplitudes of fluctuating barotropic currents. However, the information in Nohr and Gustafsson (2009) is not sufficiently detailed to allow extraction of figures of energy transfer for the Bornholm Sea.

We found that the oxygen consumption below 85 m in the Bornholm Basin doubled from the 1970s to the period 2000–2009 and noted that this is in line with the trend of increasing frequency of anoxia from the end of the 1950s to the present seen in Fig. 2. The trend of increasing oxygen consumption is most probably not a regional phenomenon but it seems to have occurred in the whole Baltic proper. Eilola (1998) used oxygen budgets to estimate oxygen consumption beneath the surface layer in the Baltic proper. He found that the consumption doubled from the 1930s to the 1980s. Sandén and Håkansson (1996) found that the observed decrease of the Secchi depth from 1919–1939 to 1969–1991, indicates a doubling of both primary production and net production since the 1930s. An analysis of the reason for these changes is outside the scope of the present paper. However, it seems that land-based sources of nutrients are generally assumed to be the cause of eutrophication, e.g., Conley (2012).

The horizontal area of the deepwater in the Bornholm Basin is about 1/10 of that of the whole Baltic proper. One would then roughly expect that the oxygen supply needed to avoid anoxic bottoms in the Bornholm Basin would be about 10 % of that in the whole Baltic proper. Stigebrandt and Gustafsson (2007) estimated that the oxygen need for the whole Baltic proper to avoid anoxic bottoms could be covered by the oxygen in about 10 000 m^3^ s^−1^ of oxygen saturated winter water. The need of winter water, 800 m^3^ s^−1^, to oxygenate the Bornholm Basin estimated in this paper fits nicely with that estimate.

The description of entrainment into jets and buoyant plumes caused by pumping was deliberately simplified in the present application. It is, however, easy to implement dynamical models for both the jet and plume mixing, e.g., Fischer et al. (1979).

The so-called reproduction volume for cod in the Bornholm Basin, defined by *S* > 11, O_2_ > 2 mL L^−1^, and *T* > 1.5 °C, varies strongly between different years (Hinrichsen et al. 2007). The reproduction volume will most likely increase when the oxygen conditions and the vertical stratification in the Bornholm Basin are changed by pumping. Oxygenation will also lead to colonization of bottoms that are presently dead. These and other ecological consequences of pumping will be reported elsewhere.

## References

[CR1] Axell LB (1998). On the variability of Baltic Sea deepwater mixing. Journal of Geophysical Research.

[CR2] Conley DJ (2012). Save the Baltic Sea. Nature.

[CR3] Eilola, K. 1998. Oceanographic studies of the dynamics of freshwater, oxygen and nutrients in the Baltic Sea, PhD Thesis. Earth Sciences Centre Publ. A30, ISSN 1400-3813, Gothenburg, Sweden: University of Gothenburg.

[CR4] Fischer, H.B., J.E. List, C.R. Koh, J. Imberger, and N.H. Brooks. 1979. *Mixing in inland and coastal waters*, 483 pp. New York: Academic Press.

[CR5] Gargett AE (1984). Vertical eddy diffusivity in the ocean interior. Journal of Marine Research.

[CR7] Gustafsson, B.G. 2003. A time-dependent coupled-basin model of the Baltic Sea. Report no. C47, Earth Sciences Centre. Göteborg University, Göteborg, 61 pp.

[CR8] Gustafsson BG, Andersson HC (2001). Modeling the exchange of the Baltic Sea from the meridional atmospheric pressure difference across the North Sea. Journal of Geophysical Research.

[CR28] Gustafsson, B.G., and A. Stigebrandt. 2007. Dynamics of nutrients and oxygen/hydrogen sulphide in the Baltic Sea deepwater. *Journal of Geophysical Research* 112: G02023. doi:10.1029/2006JG00030.

[CR9] Hinrichsen H-H, Voss R, Wieland K, Köster F, Andersen KH, Margonski P (2007). Spatial and temporal heterogeneity of the cod spawning environment in the Bornholm Basin, Baltic Sea. Marine Ecology Progress Series.

[CR10] Holtermann, P.L., L. Umlauf, O. Schmale, G. Rehder, T. Tanhua, and J. Waniek. 2011. The Baltic Sea Tracer Release Experiment. Part I: Mixing rates. *Journal of Geophysical Research* – *Oceans.* 117: C01021. ISSN 0148-0227. doi:10.1029/2011JC007439.

[CR11] Holtermann PL, Umlauf L (2012). The Baltic Sea Tracer Release Experiment: 2. Mixing processes. Journal of Geophysical Research.

[CR13] Lass HU, Mohrholz V, Seifert T (2001). On the dynamics of the Pomeranian Bight. Continental Shelf Research.

[CR14] Liljebladh, B., and A. Stigebrandt. 2000. The contribution from the surface layer via internal waves to the energetic of deepwater mixing in the Baltic. In *Experimental studies of some physical oceanographic processes,* ed. B. Liljebladh, PhD Thesis. Earth Sciences Centre, Publ. A56, ISSN 1400-3813. Gothenburg, Sweden: University of Gothenburg.

[CR15] Meier HEM (2001). On the parameterization of mixing in three-dimensional Baltic Sea models. Journal of Geophysical Research.

[CR16] Nohr C, Gustafsson BG (2009). Computations of energy for diapycnal mixing in the Baltic Sea due to internal wave drag acting on wind-driven barotropic currents. Oceanologia.

[CR17] Omstedt, A. 2011. *Guide to process based modeling of lakes and coastal seas*, 258 pp. Berlin: Springer. doi:10.1007/978-3-642-17728-6.

[CR18] Sandén P, Håkansson B (1996). Long-term trends in Secchi depth in the Baltic Sea. Limnology and Oceanography.

[CR19] Schinke H, Matthäus W (1998). On the causes of major Baltic inflows—an analysis of long time series. Continental Shelf Research.

[CR20] Seifert, T., F. Tauber, and B. Kayser. 2001. A high resolution spherical grid topography of the Baltic Sea, revised version. Paper presented at Baltic Sea Science Congress, November 25–29, Stockholm, Sweden, Leibniz-Inst. für Ostseeforschung, Warnemünde, Germany. www.io-warnemuende.de.

[CR21] Stigebrandt A (1976). Vertical diffusion driven by internal waves in a sill fjord. Journal of Physical Oceanography.

[CR22] Stigebrandt A (1987). Computations of the flow of dense water into the Baltic Sea from hydrographical measurements in the Arkona Basin. Tellus.

[CR23] Stigebrandt A (1987). A model for the vertical circulation of the Baltic deep water. Journal of Physical Oceanography.

[CR25] Stigebrandt, A. 2012. Hydrodynamics and circulation of fjords. In *Encyclopedia of lakes and reservoirs*, ed. L. Bengtsson, R.W. Herschy, and R.W. Fairbridge. doi:10.1007/978-1-4020-4410-6.

[CR26] Stigebrandt A, Aure J (1989). Vertical mixing in basin waters of fjords. Journal of Physical Oceanography.

[CR29] Stigebrandt, A., and B.G. Gustafsson. 2007. Improvement of Baltic proper water quality using large-scale ecological engineering. *AMBIO* 36: 280–286. 10.1579/0044-7447(2007)36[280:iobpwq]2.0.co;2 17520945

[CR27] van der Lee EM, Umlauf L (2011). Internal wave mixing in the Baltic Sea: Near-inertial waves in the absence of tides. Journal of Geophysical Research.

